# Proposed public policies to improve outcomes in vascular surgery: an experts’ forum

**DOI:** 10.31744/einstein_journal/2023AE0241

**Published:** 2023-08-03

**Authors:** Nelson Wolosker, Andressa Cristina Sposato Louzada, Felipe Soares Oliveira Portela, Marcelo Fiorelli Alexandrino da Silva, Guilherme de Paula Pinto Schettino, Lucas Hernandes Corrêa, Edson Amaro Juniordata, Marcelo Passos Teivelis

**Affiliations:** 1 Hospital Israelita Albert Einstein São Paulo SP Brazil Hospital Israelita Albert Einstein, São Paulo, SP, Brazil.; 2 Faculdade Israelita de Ciências da Saúde Albert Einstein Hospital Israelita Albert Einstein São Paulo SP Brazil Faculdade Israelita de Ciências da Saúde Albert Einstein, Hospital Israelita Albert Einstein, São Paulo, SP, Brazil.

**Keywords:** Big Data, Vascular surgical procedures, Carotid artery diseases, Peripheral arterial disease, Endovascular procedures, Vascular diseases, Amputation, surgical, Public Policy, Health Policy

## Abstract

**Objective:**

To evaluate outcomes of vascular surgeries and identify strategies to improve public vascular care.

**Methods:**

This was a descriptive, qualitative, and cross-sectional survey involving 30 specialists of the *Hospital Israelita Albert Einstein* via Zoom. The outcomes of vascular procedures performed in the Public Health System extracted through Big Data analysis were discussed, and 53 potential strategies to improve public vascular care to improve public vascular care.

**Results:**

There was a consensus on mandatory reporting of some key complications after complex arterial surgeries, such as stroke after carotid revascularization and amputations after lower limb revascularization. Participants agreed on the recommendation of screening for diabetic feet and infrarenal abdominal aortic aneurysms. The use of Telemedicine as a tool for patient follow-up, auditing of centers for major arterial surgeries, and the concentration of complex arterial surgeries in reference centers were also points of consensus, as well as the need to reduce the values of endovascular materials. Regarding venous surgery, it was suggested that there should be incentives for simultaneous treatment of both limbs in cases of varicose veins of the lower limbs, in addition to the promotion of ultrasound-guided foam sclerotherapy in the public system.

**Conclusion:**

After discussing the data from the Brazilian Public System, proposals were defined for standardizing measures in population health care in the area of vascular surgery.

## Highlights

Notification of complications of arterial surgeries is essential in identifying strategies to improve surgical outcomes.

Screening of prevalent and/or morbid diseases allows early intervention and prevention of complications.

Use of telemedicine in vascular follow-up allows optimizing the use of resources and reducing the burden on health services.

Concentrating complex cases in reference hospitals leads to improved surgical outcomes.

**Figure:**
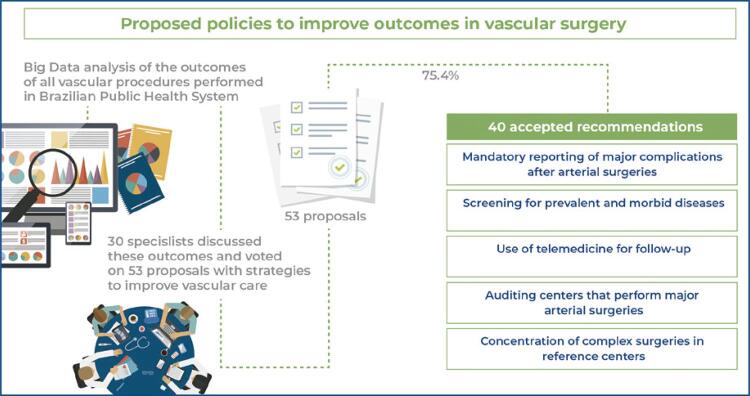

## INTRODUCTION

In recent decades, great advances in technology and medicine have significantly reduced the morbidity and mortality of several diseases. Unfortunately, this improvement is not uniform, as low- and middle-income countries still face many challenges that have already been overcome in high-income countries. These include unfavorable surgical outcomes with high rates of disabilities and preventable deaths.^(1)^

Knowing the local epidemiology of surgical outcomes (number of procedures performed, their trends, mortality, and costs) will help develop robust and efficient strategies to improve surgical outcomes. This requires complex and reliable population-based data outside the artificially controlled environments of clinical trials that will reflect the real quality of health services and expose the main aspects needing improvement.

To obtain this data, studies on vascular surgery dealing with Big Data analysis in health care were conducted. They used large volumes of data that were publicly available in the TabNet platforms of the Informatics Department of the Unified Health System (DATASUS - *Departamento de Informática do Sistema Único de Saúde*)^(2)^ and *Fundação Oswaldo Cruz* (Fiocruz) were evaluated.^(3)^ The data in these platforms are anonymized and must be entered by accredited public hospitals of the Unified Health System (SUS - *Sistema Único de Saúde*) to receive reimbursement for provided health services. These studies were combined to construct an epidemiological panorama of vascular surgery procedures and some of their main outcomes for the city of São Paulo, the state of São Paulo, the regions, and the entire nation.

In various scenarios, the study of Big Data in healthcare is helping to plan and execute strategies to improve patient care and create value in healthcare organizations.^(4)^

In Brazil, the first step has been taken, and epidemiological analyses of carotid surgeries,^(5,6)^ aortic surgeries,^(7–10)^ peripheral arterial disease (PAD),^(11)^and chronic venous disease (CVD)^(12)^ have already been published.

To propose strategies for improving the Brazilian public health care, together with the clinical staff, vascular surgeons, and interventional radiologists of *Hospital Israelita Albert Einstein*, we created the DATASUS Forum.

## OBJECTIVE

To evaluate outcomes of various vascular surgeries and identify possible standardized measures (when there was a consensus) for improving public vascular care.

## METHODS

This study was a descriptive, qualitative, and cross-sectional survey. Vascular surgeons, interventional radiologists, and members of the clinical staff of *Hospital Israelita Albert Einstein* received an invitation letter to participate in the research. Those who accepted and signed the informed consent form received relevant articles before the date of the event were included in this study. The study was approved by the hospital’s institutional review board (CAAE: 53408621.9.0000.0071; # 5.207.441).

The DATASUS Forum was held on March 19, 2022, at *Hospital Israelita Albert Einstein* in the city of São Paulo (Brazil), with online broadcasting via Zoom (Zoom Video Communications–San Jose, CA, EUA).

Thirty specialists (90% vascular surgeons and 10% interventional radiologists) attended the Forum. During the event, a theoretical exposition of the research-based epidemiology of aortic and carotid vascular surgeries, PAD, lower limb amputation, and CVD of the lower limbs in Brazil, in the state of São Paulo, and in the city of São Paulo, was presented.

After this presentation, 53 proposals illustrating potential strategies to improve public care and vascular surgery were discussed and voted on. Participants were asked to respond to each proposition with “yes” (when they agreed with it), “no” (when they disagreed with it), or “indifferent.”

The answers were collected anonymously utilizing polls via Zoom. A minimum agreement percentage of 80% was considered a consensus.

## RESULTS

Tables 1 and 2 summarize all 53 proposals and the agreement percentage for each proposal. Proposals for which there was a consensus are shown in table 1. Proposals for which there was no consensus are shown in table 2. There was consensus on 40/53 (75.4%) proposals.

**Table 1:** Proposals for which there was consensus and the percentage of agreement for each

	Consensual Proposals	Agreement* (%)
Carotid SP	1. Should the costs associated with carotid angioplasty material (stent and filter) be reduced to make it more financially viable?	90
	2. Should centers be audited regarding indications and outcomes (mortality and stroke) of carotid stenosis treatment to have better results?	100
	3. Should stroke during hospitalization and after carotid revascularization be of compulsory notification and thus included in DATASUS?	86
Carotid BR	4. Should the costs associated with carotid angioplasty material (stent and filter) be reduced to make it more financially viable?	93
	5. Should centers be audited regarding indications and outcomes of carotid stenosis treatment (mortality and stroke) to have better results?	100
	6. Should stroke during hospitalization and after carotid revascularization be of compulsory notification and thus included in DATASUS?	89
PAD SP	7. Should angioplasty remain the most commonly used technique in São Paulo, since it has the same cost and lower mortality?	81
	8. Should centers be audited on the outcomes of PAD treatment (mortality, limb salvage, and re-intervention) so that the results improve?	96
	9. Should telemedicine consulting services provided by vascular surgeons to physicians in cities with low numbers of specialists be planned?	93
	10. Should amputation be a mandatory reporting outcome after lower limb revacularization in DATASUS?	81
PAD BR	11. Should centers be audited on the outcomes of PAD treatment (mortality, limb salvage, and re-intervention) so that the results improve?	96
	12. Should amputation be a mandatory reporting outcome after lower limb revascularization in DATASUS?	81
AMPUT SP	13. Should there be a statewide program to encourage lower limb revascularization to reduce major amputation rates?	85
	14. Should there be a statewide diabetic foot screening and education program for patients and health care professionals?	100
	15. Should there be a statewide multidisciplinary care program for diabetic feet with ulcers?	100
	16. Should there be a statewide foot care education program for patients with PAD and *diabetes mellitus* during the fall to avoid the spikes in amputations seen in winter?	85
TEVAR SP	17. Should there be a follow-up control program for patients with acute aortic syndrome who were clinically treated in the emergency room?	88
	18. Should centers be audited on the outcomes of thoracic aortic treatment (mortality and re-intervention) so that the results improve?	100
	19. Should referral centers for TEVAR be created in other states, so patients are treated closer to their residence?	92
TEVAR BR	20. Should there be a follow-up control program for patients with acute aortic syndrome who were clinically treated in the emergency department?	92
	21. Should centers be audited on the outcomes of thoracic aortic treatment (mortality and re-intervention) so that the results improve?	100
IRAAA SP	22. Should there be any effort to reduce the cost of endovascular materials?	96
	23. Should centers be audited for IRAAA treatment outcomes (mortality and re-intervention) to improve results?	100
	24. Should there be a telemedicine follow-up program for IRAAA?	84
	25. Should there be a screening program for abdominal aneurysmal disease to decrease the number of emergency surgeries in São Paulo?	88
IRAAA BR	26. Should there be a national screening program for abdominal aneurysmal disease to decrease the number of emergency surgeries in Brazil?	88
	27. Should centers be audited on the outcomes of AAAIR repair (mortality and re-interventions) so that the results are increasingly better?	96
	28. Should there be any effort to reduce the cost of endovascular materials?	96
TAA SP	29. Should there be a program to control the follow-up of thoraco-abdominal aneurysmal disease to reduce the number of emergency surgeries in São Paulo?	100
	30. Should there be more centers outside the city of São Paulo to treat this type of disease?	85
	31. Should there be a concentration of surgical cases (especially elective) in specialized centers to improve outcomes for elective surgery?	100
	32. Should there be a telemedicine follow-up program for thoracoabdominal aneurysmal disease?	85
	33. Should centers be audited on the outcomes of TAA treatment (mortality) so that the results are increasingly better?	100
TAA BR	34. Should there be a program of follow-up control of thoracoabdominal aneurysmal disease to reduce the number of emergency surgeries in Brazil?	100
	35. Should there be a telemedicine follow-up program for thoracoabdominal aneurysmal disease?	92
	36. Should centers be audited on the outcomes of TAA repair (mortality) so that the results are increasingly better?	100
	37. Should public hospitals offer endovascular treatment for thoracoabdominal aneurysms paid for by SUS?	84
CVD SP	38. Should the Unified Health System also invest in other techniques, such as echoguided sclerotherapy?	88
	39. Should there be a greater incentive to operate on varicose veins of both limbs in the same anesthetic-surgical procedure?	85
CVD BR	40. Should the Brazilian Public Health System also invest in other techniques, such as the use of echoguided sclerotherapy?	96

BR: Brazil; SP: state of São Paulo; PAD: peripheral arterial disease; AMPUT: amputation; TEVAR: thoracic endovascular aortic repair; IRAAA: infrarenal abdominal aortic aneurysm; TAA:thoracoabdominal aortic aneurysm; CVD: chronic venous disease.

**Table 2:** Proposals for which there was no consensus and the percentage of agreement for each

	Non-consensual proposals	Agreement* (%)
CAROTID SP	1. Should carotid endarterectomy be the most commonly used technique in São Paulo, since it is cheaper, has similar results and the same mortality with other procedures?	72
CAROTID BR	2. Should carotid endarterectomy be Brazil’s most commonly used technique since it is cheaper, despite the higher mortality?	63
PAD BR	3. Should angioplasty remain the most commonly used technique for lower limb revascularization in Brazil since the mortality is lower, despite the higher costs?	77
TEVAR SP	4. Should there be a city and state screening program for thoracic aortic diseases to reduce the number of emergency surgeries?	58
TEVAR BR	5. Should there be a national screening program for thoracic aortic diseases to reduce the number of emergency surgeries?	65
IRAAA SP	6. If the price of endovascular materials were reduced to 30% of what they cost today, should endovascular technique be the technique of choice in São Paulo for all IRAAA with favorable anatomies?	68
IRAAA BR	7. If the price of endovascular materials were reduced to 30% of what they cost today, should the endovascular technique be the technique of choice in Brazil for all IRAAAs with favorable anatomies?	71
	8. Should there be a telemedicine follow-up program for IRAAA?	79
TAA SP	9. Should there be a municipal and state screening program for thoracoabdominal aneurysmal disease to reduce the number of emergency TAA repairs in São Paulo?	73
TAA BR	10. Should there be a national screening program for thoracoabdominal aneurysmal disease to reduce the number of emergency TAA repairs in Brazil?	68
CVD SP	11. Should the Brazilian Public Health System also invest in endovenous thermoablation for the treatment of varicose veins?	58
CVD BR	12. Should the Brazilian Public Health System also invest in other techniques, such as thermoablative techniques for the treatment of varicose veins?	54
	13. Should there be a program to encourage the surgical treatment of varicose veins on a national level?	79

BR: Brazil; SP: state of São Paulo; PAD: peripheral arterial disease; AMPUT: amputation; TEVAR: thoracic endovascular aortic repair; IRAAA: infrarenal abdominal aortic aneurysm; TAA: thoracoabdominal aortic aneurysm; CVD: chronic venous disease.

## DISCUSSION

Of 53 public policy proposals voted on in this study, the expert panel reached a consensus on 40 proposals involving notification of diseases, screening of prevalent conditions, and concentration of complex cases in reference centers.

Expert opinion/consensus is used in research that answers the commonest questions in clinical practice, *i.e*., real-life situations that are often excluded in randomized controlled trials because they fall outside the strict inclusion criteria.

Our institution performed a prior consensus,^(13)^ and this one was partially based on it. However, instead of using clinical cases, we used real-world data and identified proposals for improving public health care in vascular surgery based on consensus.

We discussed seven major vascular interventions for different diseases based on the participants’ responses on the following aspects: notification of diseases, screening, use of telemedicine for follow-up, auditing of results, creation of reference centers for highly complex surgeries, costs of materials, and treatment of lower limb varicose veins.

## NOTIFICATION

Compulsory notification of diseases in vascular surgery is a relevant and detailed indicator of the quality of health care in a population. It allows identification of areas needing improvement, leading to actions from the primary (*e.g.*, health education for diabetic foot care) to tertiary level (*e.g.*, change of peri-operative protocols for complex arterial surgeries). The participants were asked whether some conditions/situations should be compulsorily notified for better detailing.

### There was a consensus on the compulsory notification of:

#### Stroke after carotid revascularization

The occurrence of peri-procedural stroke in carotid revascularization implies high morbidity and mortality for the patient and may also reflect the quality of carotid revascularization and its peri-operative care.^(14)^ The Brazilian health system database does not allow identification and characterization of these postoperative events. With mandatory reporting, this information would be more easily accessible, allowing better data for audits and studies.

There are few studies on the etiologies of stroke after carotid revascularization. However, recent papers suggest classifying postoperative ischemic events into four main etiologies: hemorrhagic events, embolism (cardiac- or carotid-related), carotid occlusion, or hemodynamic events (hypo- or hyperperfusion). This classification is based on few clinical variables: time of the event after the carotid procedure, affected brain territory, and severity of the ischemic event.^(15)^ Knowing the real incidence of this event through a compulsory notification may be the best way to improve patient outcomes, especially with the implementation of protocols for early recognition and intervention.^(16)^

Studies on amputations after revascularization for PAD have reported that amputation rates after lower limb revascularization procedures vary between 0 and 7%.^(17,18)^ In the Brazilian health system, the registration of amputations does not allow identification of those that occur after revascularization attempts. Obtaining information on the time between revascularization and amputation, revascularization technique (open or endovascular), re-intervention rate, and amputation level (above or below the knee) is of fundamental importance to improve PAD care.^(19)^

## SCREENING

Screening is the search for abnormalities/diseases in asymptomatic persons who have an increased risk of developing the disease or its complication.^(20)^

### There was a consensus in two situations:

#### Identification of diabetic feet

Epidemiological studies report that the risk of lower limb ulcers in patients with diabetes is approximately 2.5% annually. Diabetic foot injuries are responsible for more than 100,000 amputations per year in the United States of America (USA).^(21)^ Moreover, the estimated annual cost of lower limb injuries in patients with diabetes in the USA is $9–13 billion.

Early identification of patients at risk of developing lesions is crucial in reducing these impacts on the individual (loss of quality of life and risk of amputation) and on society (high annual costs for the care of diabetic foot complications).^(22)^ Studies suggest multiple factors for selecting patients at risk for diabetes-related injuries, such as visual impairment, changes in foot sensitivity, and skin mycoses, all detectable at the primary health care level.^(23)^

Finally, in view of these findings, societal consensus recommends educating patients on diabetic foot care and patients with PAD to prevent lesions and their complications.^(24)^ In our study, there was a consensus on implementing education programs for patients and health professionals with the aim of reducing the incidence of diabetes-related and PAD-related complications.

## Aortic aneurysm screening

Screening for abdominal aortic aneurysm (AAA) can reduce the number of ruptures and high-mortality urgency/emergency AAA repairs, thus preventing AAA-related deaths.^(25)^ The Brazilian Society of Angiology and Vascular Surgery recommends AAA screening with abdominal ultrasonography for individuals aged 65 to 75 years with a smoking history.^(26)^ However, the Brazilian Ministry of Health does not recommend same. Previous studies have highlighted the burden of AAA mortality in the Brazilian population, especially in older individuals. These studies have also stated the need for a plan to prevent AAA-related deaths.^(27)^ Our experts agreed on municipal and national plans.

Regarding the screening for thoracic and thoracoabdominal aortic aneurysms, the agreement was <80% in this study, consistent with existing studies, because there is still no evidence of the benefit for screening for aneurysmal disease involving the thoracic aorta.^(28)^ Such a measure is not cost-effective because of the relatively low prevalence of this disease and because there are no less invasive and less expensive tests to perform this screening, unlike AAA screening that can be done with abdominal ultrasonography.^(29,30)^

## TELEMEDICINE

During the coronavirus disease (COVID-19) pandemic, there was an increase in the use of telemedicine. Initially, this tool was used to allow access to health services at a time when it was important to maintain social isolation as a measure to prevent viral transmission.^(31)^However, the use of telemedicine improved and expanded after the pandemic was controlled and health services began to evaluate the ethical, legal, and social aspects of this type of care.^(32)^

There was consensus on the following indications for remote consultation with a vascular surgeon: follow-up for descending thoracic aortic dissections that have been treated clinically during the acute presentation, thoracoabdominal aortic aneurysms and AAAs with a diameter below 5cm.

Existing studies have assessed the use of telemedicine in vascular surgery, especially in aortic diseases.^(33,34)^ Telemedicine can also be used for the follow-up of asymptomatic patients and patients with no surgical indication who need regular follow-up (*e.g*., patients with small-diameter aneurysms). This may allow monitoring of these patients at the primary health care level, with remote follow-up by a specialist physician, without needing an in-person visit. With this, tertiary and quaternary services can be “unburdened” and efforts (and expenses) focused on more complex situations with surgical indications.

## CENTER AUDIT

There was consensus that the outcomes of endovascular and open procedures for carotid and lower limb revascularizations, aortic repairs (aneurysms and dissections), and high morbidity and mortality arterial surgeries should be audited at each center. This is possible using TabNet, as the municipal data can be extracted according to their distributions per services.

Regarding carotid revascularization, the perioperative risk of stroke and death in asymptomatic patients undergoing carotid procedures should be less than 3% to ensure benefit from the surgical approach.^(35)^ Understanding the outcomes of carotid surgery is the basis for developing strategies to improve outcomes.

Regarding lower limb revascularization, data on mortality, re-intervention, and major and minor amputation rates are essential to inform therapeutic decisions in terms of surgical technique, postoperative care, and rehabilitation.^(36,37)^

Regarding aortic repair, a highly complex surgery with severe potential complications,^(38)^ knowing the outcomes is essential in reducing postoperative mortality. For example, in the United Kingdom, after an epidemiological analysis revealed that the local mortality rate following elective AAA open repair was worse than that in neighboring countries, an initiative was implemented to evaluate and change perioperative care, especially anesthetic care. In approximately 4 years, it was possible to reduce the mortality to less than one-third of the initial mortality.^(39)^

## REFERENCE CENTERS

There was a consensus to concentrate complex surgeries in reference centers for thoracic and thoracoabdominal aortic surgery.

The relationship between higher case volume in a service/surgeon and better patient outcomes in complex surgeries has been extensively investigated.^(40)^

A recent study in South Korea showed that this relationship is also valid in complex thoracic aortic surgeries. In-hospital mortality in high-volume centers (>60 cases/year) was 8.6%, while the mortality in low-volume centers (<30 cases/year) reached 21.9%.^(41)^

Our expert forum also suggested creating new referral centers, increasing the capillarity of services, reducing travel, and facilitating patient access.

## COSTS OF MATERIALS

There was a consensus that the cost of endovascular materials for carotid revascularization and aortic aneurysm repair should be reduced.

Endovascular materials usually account for most of the costs of this type of procedure,^(42)^ with stent costs ranging from $8,100 to $28,200. In cases of endovascular repair of uncomplicated AAA, the cost of a stent graft may represent 52% of the total expenses of the procedure.^(43)^

Some studies report the reduction of expenses with stent grafts for aortic aneurysms by up to 30.8%, from the restructuring of contracts for the acquisition of materials, comparison of prices in the market, and transparency in negotiations with suppliers.^(44)^

Thinking about strategies at a national level, stimuli such as tax incentives or the development of a structure that would allow national manufacturing may help reduce the costs of endovascular materials, allowing greater diffusion of these techniques.

## LOWER LIMB VARICOSE VEINS

There was consensus on encouraging simultaneous treatment of both limbs and using ultrasound-guided foam sclerotherapy to treat CVD in the public system.

On the other hand, there was no consensus on investing in endovenous ablative techniques.

There is no statistical difference in pain, return to work activities, return to physical activities, or aesthetic result between unilateral and bilateral surgical treatment of varicose veins.^(45)^ However, patients report better quality of life with staged treatment.^(46)^Considering public expenditure, the simultaneous bilateral approach instead of a sequential unilateral one may have lower costs of hospitalization, anesthetic procedures, and time away from work activities.

Ultrasound-guided foam sclerotherapy is a safe and effective alternative for treating CVD of the lower limbs.^(47)^A review of randomized, controlled studies comparing thermal ablation and echoguided sclerotherapy methods demonstrated that although there is a superior success rate for the ablative techniques from an anatomical standpoint (complete obliteration of the vein), clinical success and patient outcomes are similar between the two techniques. There is also no difference in morbidity and complication rates. In addition, sclerotherapy has a significantly lower cost than ablative techniques.^(48)^ This may explain the participants’ choice of promoting ultrasound-guided foam sclerotherapy over endovenous thermoablative techniques.

## CONCLUSION

From this real-world data review, with a better understanding of the reality in Brazil, in the city, and in the state of São Paulo, proposals were made for improving population health care in the area of vascular surgery.
